# Supplementary material for: COZOID: contact zone identifier for visual analysis of protein-protein interactions
Source: BMC Bioinformatics. 2018 Apr 6;19:125. doi: 10.1186/s12859-018-2113-6 (PMC5889581; doi:10.1186/s12859-018-2113-6)
Supplement: Supplementary file 2 — Software build. Executable binary file of the software tool. (ZIP 118,784 kb) [file 12859_2018_2113_MOESM2_ESM.zip › cozoid/platform/docs/testng-6.5.1-javadoc.zip!/org/testng/annotations/Guice.html]

Guice (TestNG)


---


|  |  |  |
| --- | --- | --- |
| **Overview** \| **Package** \| **Class** \| **Tree** \| **Deprecated** \| **Index** \| **Help** | |  |
| **PREV CLASS**   **NEXT CLASS** | **FRAMES**    **NO FRAMES**     **All Classes** |  |
| SUMMARY: REQUIRED \| OPTIONAL | DETAIL: ELEMENT |  |


---


## org.testng.annotations Annotation Type Guice

---

``` @Retention(value=RUNTIME) @Target(value=TYPE) public @interface Guice ```

This annotation specifies what Guice modules should be used to instantiate
this test class.

**Author:**
:   Cedric Beust

---

| **Optional Element Summary** | |
| --- | --- |
| `java.lang.Class<? extends IModuleFactory>` | `moduleFactory` |
| `java.lang.Class<? extends com.google.inject.Module>[]` | `modules` |

### modules

```
public abstract java.lang.Class<? extends com.google.inject.Module>[] modules
```

:   **Returns:**: the list of modules to query when trying to create an instance of this test class.

    **Default:**: {}

---


### moduleFactory

```
public abstract java.lang.Class<? extends IModuleFactory> moduleFactory
```

:   **Default:**: org.testng.IModuleFactory.class


---


|  |  |  |
| --- | --- | --- |
| **Overview** \| **Package** \| **Class** \| **Tree** \| **Deprecated** \| **Index** \| **Help** | |  |
| **PREV CLASS**   **NEXT CLASS** | **FRAMES**    **NO FRAMES**     **All Classes** |  |
| SUMMARY: REQUIRED \| OPTIONAL | DETAIL: ELEMENT |  |


---
